# Assessing risks for bovine and zoonotic tuberculosis through spatial analysis and a questionnaire survey in Fiji – A pilot study

**DOI:** 10.1016/j.heliyon.2023.e22776

**Published:** 2023-11-23

**Authors:** Jenny-Ann L.M.L. Toribio, Keresi Lomata, Sam Fullman, Aaron Jenkins, Elva Borja, Shumaila Arif, Jarrad McKercher, David Blake, Anabel Garcia, Richard J. Whittington, Frank Underwood, Ben J. Marais

**Affiliations:** a Sydney School of Veterinary Science, Faculty of Science, The University of Sydney, NSW, Australia; b Ministry of Agriculture, Koronivia Research Station, Koronivia, Republic of Fiji; c Ministry of Health and Medical Services, Dinem House, 88 Amy Street, Suva, Republic of Fiji; d Sydney Infectious Diseases Institute (Sydney ID), The University of Sydney, NSW, Australia; e Edith Cowan University, Centre for People, Place and Planet, 270 Joondalup Drive, Joondalup, WA, Australia

**Keywords:** Extra pulmonary tuberculosis, Bovine tuberculosis, *Mycobacterium bovis*, Zoonotic tuberculosis, One health, Fiji

## Abstract

*Mycobacterium bovis* causes tuberculosis in cattle and when transmitted to humans typically causes extra-pulmonary tuberculosis (EPTB). Bovine tuberculosis (bTB) has a global distribution and is controlled in most countries to protect animal and public health. Recent studies revealed that bTB is established on dairy farms in Fiji where EPTB cases have been reported in people. The aims of this pilot investigation were to look for putative zoonotic TB (EPTB) cases in people and to evaluate practices that might contribute to the persistence and transmission of *M. bovis* between cattle and to humans. Existing data sets were shared between the Fiji Ministry of Agriculture and Ministry of Health and a questionnaire-based survey was implemented using One Health principles. Statistically significant co-location and close proximity of EPTB cases and bovine TB affected farms were identified. The bTB infection status of farms was significantly associated with unfenced water sources where cattle grazed. Of 247 households, 65 % shared drinking water sources with cattle and 36 % consumed raw milk without boiling, while 62 % of participants reported backyard slaughter of cattle. Several participants reported current symptoms potentially suggestive of TB (chronic cough) but the impact of smoking and history of previous TB treatment could not be evaluated. Farmers had limited understanding of the practices required to prevent bTB at farm level. Further study is recommended and should include an assessment of lifetime EPTB diagnoses, classification of farms based on more recent bTB test data and molecular typing of mycobacterial isolates from humans, cattle and the environment. A targeted awareness and education approach is required to reduce the future risk of zoonotic TB and to help ensure uptake of recommendations and practices aimed at controlling and preventing bTB.

## Highlights

• A One Health study and analysis of TB in cattle and people.
• First study to map co-located human EPTB cases and bovine TB infected cattle herds in Fiji.
• Risk factors were identified for *M. bovis* transmission to humans.
• Farmers had limited understanding of ways to prevent bovine TB.

## 1 Introduction

Bovine tuberculosis (bTB) is a chronic infectious *disease of livestock caused by Mycobacterium bovis.* It occurs in both intensive and extensive husbandry systems [1] and is responsible for serious economic losses due to livestock disposal, carcass confiscation, premature culling, low production and poor reproductive performance [2]. In developing countries this affects livelihoods by compromising sustainable food supply, income and social status [3,4]. *M. bovis* can be transmitted to humans, particularly where bTB prevention and control measures are weak [5], and it typically causes extra-pulmonary tuberculosis (EPTB) [6]. It is estimated that around 150,000 new cases of zoonotic TB occur in people leading to 12,500 deaths globally every year [7]. However, such estimates are imprecise because zoonotic TB goes largely unreported due to poor public health surveillance in developing countries where most cases occur. In this paper we use World Health Organisation (WHO) terminology, which considers “zoonotic TB” to be disease caused by *M. bovis* infection in people and “bovine TB” to be disease caused by *M. bovis* infection in animals [7]. *M. bovis* can be transmitted to humans through consumption of raw milk and direct contact with livestock [8] leading to EPTB, but inhalation leading to pulmonary TB is also possible [5].

Bovine TB has a global distribution and because of its impacts on cattle production, the zoonotic risk and regulations on transboundary trade, measures aimed at control and eradication of the disease are mandated by the World Organisation for Animal Health [9] and are observed in many countries. In Fiji, where there has been a bovine TB control program for decades, the disease continues to be a focus for the Government. However, testing and culling of *M. bovis* infected cattle is not uniformly welcomed by farmers because it negatively impacts productivity even with financial compensation from the government [10]. A retrospective study from 1999 to 2014 reported that bovine TB was well established on dairy farms in Fiji, because of limited diagnostic testing, use of outdated diagnostic tests, lack of field staff training, uncontrolled cattle movements, lack of standard procedures for bovine TB data collection and the presence of wild/stray cattle [10]. More recent analysis for the period 2015–2020 reconfirmed the high prevalence of bTB [11]. There is limited information about the practices in livestock farming communities that could influence the transmission of *M. bovis* to cattle and humans, and there are no curated data on zoonotic TB in Fiji. However, in 2013, 71 cases of EPTB out of a total 269 TB cases (26 %) were notified in Fiji [12], while in 2016, 29 % of 312 cases of TB were EPTB [13], suggesting that *M. bovis* could be involved. More detailed, long-term information is essential to understand the factors that contribute to the persistence of *M. bovis* in cattle and to reduce exposure pathways for zoonotic TB.

The aims of this pilot investigation were to look at the potential contribution of *M. bovis* to human TB (especially EPTB) cases in Fiji and to evaluate high risk practices for local transmission of *M. bovis*. Firstly, using existing data, the overlap in the geographical distribution of EPTB cases in humans and bovine TB in cattle was studied to identify locations that may present a high risk for zoonotic TB. Secondly, in these locations, high risk practices for *M. bovis* transmission between cattle and to humans were studied using a questionnaire-based survey. This initial research sought to improve understanding about the persistence of bovine TB in cattle and avenues for transmission to people, and to direct further research, consistent with the WHO's call to action in its Roadmap for Zoonotic Tuberculosis [7]. It also served as a first step to facilitate research collaboration on zoonotic TB for the Ministry of Health and Medical Services (MHMS) and the Ministry of Agriculture (MOA) in Fiji through the sharing of datasets and designing and implementing a joint investigation using One Health principles, which are discussed below.

## 2 Material & methods

### 2.1 Retrospective analysis of TB cases among cattle herds and people

#### 2.1.1 Study area and datasets

The study area for this risk-based investigation was the two provinces of Naitasiri and Tailevu in Central Division, Fiji which have received the most intense surveillance for bovine TB by the Ministry of Agriculture (MOA) and where there is evidence that bovine TB (bTB) is well-established in dairy herds [10,11]. These are two of 14 provinces in Fiji.

In 2020 there were 24,777 cattle (67 % dairy, 33 % beef) in Naitasiri and Tailevu, representing 75 % of the cattle in Central Division and 21 % of cattle in Fiji [14]. In 2017 the prevalence of bTB was 22.2 % in Naitasiri, and 33.7 % in Tailevu [10,11].

In 2017, the human population of these two provinces was 242,230 (177,678 in Naitasiri and 64,552 in Tailevu) out of a total population of 884,887 in Fiji [15]. In 2020, there were 1245 and 2084 households engaged in raising livestock in Naitasiri and Tailevu, respectively, and there were 51,270 agricultural household members >10 years old in these provinces [14,16], mainly indigenous Fijians (Itaukei).

Data from these human and cattle populations were obtained for a period of three years (2015–2017). The datasets used were: i) all diagnosed human TB cases categorised as EPTB, with de-identified data listing village of residence, gender and age at diagnosis; ii) all bovine TB infected cattle herds, with de-identified data listing location of farm/village (front gate or centroid), type of cattle herd (dairy, beef), number of cattle in herd at time of herd testing by the MOA, number of cattle tested and number that tested positive.

#### 2.1.2 Data analysis

All geolocation data were collected in decimal degrees and imported into Geographical Information Systems (GIS) using Universal Transverse Mercator (UTM) zone 60S with World Geodetic System (WGS) 84 datum and processed in ArcGIS Pro 3.1 (ESRI). The locations mapped were the communities of residence for EPTB cases and the farm front gate. Human cases and farm locations (feature) were mapped and investigated within tikina (clan) boundaries that relate to major family-owned land and within water sub-catchment boundaries.

A colocation analysis [17] was performed to determine if there was a spatial association between human EPTB cases and bovine TB infected herds. In the colocation analysis, each feature is evaluated individually to determine if it is co-located with neighbouring human EPTB cases or bovine TB infected herds. A local colocation quotient (LCLQ) is assigned to each feature, with values greater than 1 representing significant co-located features, values less than 1 representing significant isolated features, and intermediate values representing non-significant co-located or isolated features [17]. For a feature to be co-located, it must contain, to a certain degree, an even population of both human EPTB cases and bovine TB infected herds. Uneven distributions of either human EPTB cases and bovine TB infected herds will result in the feature being deemed isolated. The analysis was performed twice; first with human EPTB cases as the category of interest and then secondly with bovine TB infected herds as the category of interest.

To determine the features of human EPTB cases and bovine TB infected herds that were located within proximity of each other a ‘near analysis’ was performed. The ‘near analysis’ determines the spatial distance of two groups of features located within proximity to each other [18]. The analysis was used to determine the human EPTB cases that were located within 5 km of a bovine TB infected herd.

### 2.2 Survey of high-risk households

#### 2.2.1 Study area, participant selection and data collection

Households of dairy farmer and dairy farm workers with infected dairy herds in the geographic clusters identified in Tailevu and Naitasiri Provinces were selected to participate in face-to-face interviews, as were all households that owned cattle in two villages: Naloto in Tailevu and Gusuisavu in Naitasiri. Selection of these villages was based on their close proximity to known infected dairy herds and because the village cattle had bTB infected status based on testing in the previous 12 months. All those selected and invited agreed to participate in the survey. Based on data available for 2017 [11], all infected dairy farms in Naitasiri and 28 of 42 in Tailevu were included in this survey.

Each selected household was contacted by MOA BTEC staff and invited to participate in an interview. On the day of interview, the household questionnaire was completed by the household head via a face-to-face interview with a trained MOA staff member. The structured questionnaire obtained information on household demographics, history of human TB for the household, exposure to the infected cattle herd, and cattle management. Consent for the household to participate was evidenced by completion of the household questionnaire. A copy of the questionnaire is provided in Table S1. BTEC staff provided clarification on current practices to researchers when requested to do so.

For each household member, the questionnaire recorded demographics, exposure to the cattle herd, and the current presence of TB symptoms/signs. The history of human TB for the household in the previous 5 years was also recorded. The household head answered all these questions on behalf of the members in the household, which in this study, referred to all people living at the residence for the last 6 months or longer. Each household member was given a unique identification number.

For each household, the questionnaire recorded household practices relevant to potential routes of transmission from cattle to humans. In addition, for farm manager households, the household head answered questions about the grazing and feeding practices of cattle on the farm, and selection of multiple options was possible if multiple practices were in place. For the two villages, the village head completed the cattle management questions as this person had knowledge equivalent to a farm manager about the village cattle herd.

#### 2.2.2 Data management and statistical analyses

Data were entered into a Microsoft Access database and then exported to and cleaned in Microsoft Excel. For farm and household data where respondents could select multiple responses, new variables were created to accommodate these responses. For statistical analyses, the data were exported to R statistical software Version 4.0 [19] (https://www.R-project.org/.). Results of descriptive analyses at farm, household and household member levels were displayed as percentages rounded to integers (except if < 1) and counts using the *table* and *prop.table* functions in R.

##### 2.2.2.1 Farm level

The total number of cattle on each farm was calculated by adding numbers of milking cows, dry cows, heifers, bulls, steers and calves. Farms were classified based on type of operation according to herd size: a commercial farm had >40 cattle, a semi-commercial farm had 16 to 39 cattle while a subsistence farm had 15 or fewer cattle [11]. Based on the most recent BTEC testing completed on each farm, the farm TB status was classified as infected (including infected, restricted and provisionally clear) or clear using the BTEC program definitions. The association between farm TB status and farm management practices was assessed using Fisher's exact test by means of the *fisher.test* function in R due to small observed frequencies for many of the variables; maximum likelihood estimates of odds ratios were produced as part of the *fisher.test* output. The association of farm TB status with herd size (transformed as log_*e*_(size+1) due to its extreme positive skew), the only quantitative predictor, was assessed by logistic regression using the lme4 *glm* function in R [20]. Due to limited univariable associations, a multivariable model was not pursued.

##### 2.2.2.2 Household level

Three known high-risk practices for zoonotic TB transmission were investigated 1) water sources shared with cattle; 2) consumption of raw milk without boiling; and 3) backyard slaughter of cattle. The association of ethnicity and household type with each of these practices was assessed in multivariable logistic regression using the lme4 *glm* function in R [20]. Venn diagrams were constructed to visualise the number of households conducting multiple high-risk practices for each household type (farm manager, fam worker, villager) using the VennDiagram package in R.

##### 2.2.2.3 Household member level

The household head reported the presence or not of six clinical symptoms recognised to be suggestive of tuberculosis for each household member. On the basis of the reported presence of one or more of these symptoms in a household member, the binary outcome (0/1) current symptoms suggestive of TB was designated for each person and the association with demographic variables (age, gender, occupation) and household practice variables (contact with infected herd, drink raw milk, drink water used by cattle, swim/play/contact water used by cattle) was evaluated using a generalised linear mixed model with household as a random effect. The *glmer* function in the lme4 package in R was used to fit the model [20]. Due to limited statistically significant univariable associations, a multivariable model was not pursued. Comparison between the categories of each variable were assessed using CLD functions of the *emmeans* package in R. Age was the only quantitative predictor considered, and as well as a linear trend, associations were assessed using a spline to allow for the possible non-linear effect of age.

## 3 Results

### 3.1 Analysis of TB cases among cattle herds and people

**Fig. 1:**
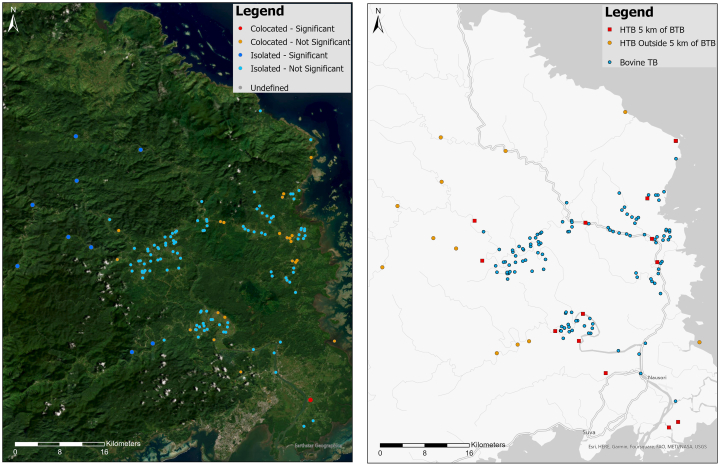
Graphical representation of the colocation analysis (left) and the ‘near analysis’ (right) showing the proximity of bovine TB and human EPTB cases in Naitasiri and Tailevu provinces of Central Division, Fiji in 2015–2017. Blue dots, bovine TB infected cattle herds; red squares human EPTB cases within 5 km of bovine TB infected cattle herds; orange dots, human EPTB cases more than 5 km from bovine TB infected cattle herds. (For interpretation of the references to colour in this figure legend, the reader is referred to the Web version of this article.)

Between 2015 and 2017, there were a total of 1032 new cases of human TB [21] and a total of 120 out of 269 dairy farms were bovine TB affected based on testing in Naitasiri and Tailevu provinces of Central Division [11]. Of these farms, verified location data were available for 114 in these two provinces. The locations of the human EPTB cases diagnosed over the 3-year study period based on their communities of residence were overlaid with the infected cattle herds based on the locations of their farm front gates. The colocation analysis determined one instance of a significant colocation of a bovine TB infected herd and human EPTB cases (Fig. 1). In this instance, bovine TB infected herds was selected as the category of interest. Additionally, in the ‘near analysis’ a visual spatial association appeared to be present as almost 50 % of EPTB cases occurred within 5 km of a known bovine TB infected farm (Fig. 1). There appeared to be a wider distribution of human EPTB cases over the Naitasiri and Tailevu provinces when compared to the bovine TB infected herds. This spatial distribution was more predominant in Naitasiri than Tailevu province and appeared to be situated around/within proximity to hydrological channels. Given the high concentration of bovine TB infected herd sample locations, the colocation analysis determined 12 colocation events but only one instance was deemed significant and the ‘near analysis’ suggests a spatial association between bovine TB infected farms and EPTB cases.

### 3.2 Questionnaire survey

#### 3.2.1 Farm level

In total, 60 farms and two villages participated in the survey. Since the villages practiced collective management of cattle in each village and thus each functioned as a farm, a total of 62 farms were evaluated. The majority (87 %) of these farms sold cow milk as their primary source of income; three (5 %) specified the sale of cattle at the annual local Magiti festival as the business purpose. In addition, 35 % of the farms used their own milk and 19 % used their own meat for household consumption.

**Fig. 2:**
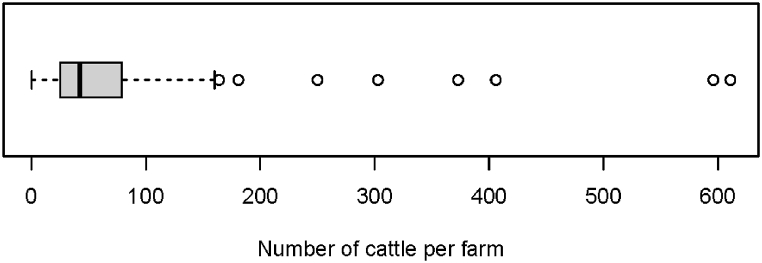
Boxplot of number of cattle per farm for 62 farms investigated in Central Division Fiji in 2018–2019 - median (vertical line), 25th and 75th percentiles (box).

Farm herd size ranged up to 611 animals, with a mean of 85 and a median of 43 (Fig. 2). Almost all farms (98 %) had fenced paddocks for animal grazing and allowed their animals to roam for grazing during part of the day (95 %) (Table 1). No farms had cattle tethered at all times. The main water source for cattle was the nearby river or creek (95 % of farms), but water troughs were also used. The majority (97 %) of farms grazed cattle in areas with a river or creek and almost half (47 %) reported that cattle owned by other people were present in the areas where their cattle graze. All farms sent their animals to abattoirs for slaughter, but 27 % also had cattle slaughter occurring on the farm and 10 % at the farmer residence.

**Table 1:** Farm management practices, farm bTB status and univariable analysis results for 62 farms investigated in Central Division Fiji in 2018–2019.

Farm management practices	Variables	No. of farms (%)^a^	Infected *No.* (%)	Clear *No.* (%)	P-value^c^	Odds ratio^d^
Cattle grazing practice	Grazing pasture in fenced paddocks	61 (98)	40 (98)	21 (100)	1	0
	Always held in stall/yard with cut and carry feeding system	7 (11)	3 (7)	4 (19)	0.21	0.34
	Tethered at all times	0				
	Roam freely for grazing	59 (95)	39 (95)	20 (95)	1	0.98
Water sources for cattle	River/creek	59 (95)	38 (93)	21 (100)	0.54	0
	Well/spring	10 (16)	7 (18)	3 (14)	1	1.23
	Dam	27 (43)	17 (41)	10 (48)	0.78	0.78
	Water trough/other container	41 (66)	26 (63)	15 (71)	0.58	0.70
Present where cattle graze	River/creek	60 (97)	39 (5)	21 (100)	0.54	0
	Unfenced dam	15 (24)	11 (27)	4 (19)	0.55	1.55
	Unfenced well or spring	9 (14)	3 (7)	6 (29)	0.05	0.20
	Cattle owned by other people	29 (47)	21 (51)	8 (38)	0.42	1.69
	Wild or unowned cattle	13 (21)	8 (20)	5 (24)	0.75	0.78
Location of slaughter	On the farm	17 (27)	9 (22)	8 (38)	0.23	0.46
	At the residence	6 (10)	4 (10)	2 (10)	1	1.03
	Abattoir	62 (100)	41 (100)	21 (100)	1	0
Farm classification following last bovine TB surveillance testing	Clear Infected^b^ Provisionally clear^b^ Restricted^b^	21 (34) 25 (40) 4 (7) 12 (19)				

a Participants could select multiple responses.

b Classified as ‘infected’; single intradermal test used as the surveillance test.

c P-value based on Fisher's exact test.

d Odds ratio based on maximum likelihood estimate.

**Fig. 3:**
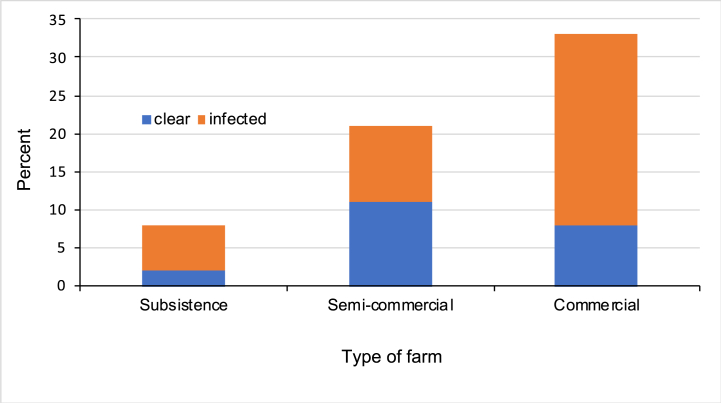
Number of *M. bovis* infected and clear cattle farms according to type of farm.

At the time of the study, 25 farms were bovine TB infected while 16 were progressing towards attaining disease-free status after previous documented infection (classified as provisionally clear or restricted). According to definitions, 41 farms were infected and 21 were considered clear (Table 1). Median herd size for infected farms was 49 compared to 39 for clear farms (P = 0.59). When classified according to type of operation, it was clear that most of the infected farms were commercial dairies (Fig. 3). However, there was no significant difference in the proportion of infected farms when farms were classified in this way (P = 0.1, Fisher's exact test). Except for an unfenced well or spring in the area where cattle graze (P = 0.05), there was no significant association between farm bovine TB infection status and any of the farm management practices evaluated (Table 1).

#### 3.2.2 Household level

Of the 247 households that participated in the study, 168 lived on dairy farms (60 farm managers and 108 farm workers) and 79 lived in the two villages Naivicula and Gusuisavu. Most households were ethnically Itaukei (78 %) and were mostly farm worker or village households (Table 2). For the 60 dairy farms, while there were almost equal numbers of ethnic Indian and Itaukei farm managers, the proportion of farm manager households among the ethnic Indian population (30/50, 62 %) was much greater than that among the Itaukei population working on these farms (28/193, 15 %).

**Table 2:** Demographics and practices related to recognised risk factors for zoonotic TB among 247 households who own or work with cattle in Central Division Fiji in 2018–2019.

Variable	Category	No. farm manager households	No. farm worker households	No. village households	Total no. households (%)^a^
Household ethnicity	Caucasian	2	2	0	4 (1.6)
	Indian	30	20	0	50 (20)
	Itaukei^b^	28	86	79	193 (78)
Main water source for household	Bore	2	0	0	2 (0.8)
	Communal dam	28	75	74	177 (72)
	Rainwater	16	13	0	29 (12)
	WAF town supply	14	20	5	39 (16)
Water sources shared with cattle	True	24	76	61	161 (65)
	False	36	32	18	86 (35)
Usual milk sources	Shop	21	32	58	111/245 (45)
	Dairy farm	8	27	2	37/245 (15)
	Equally from shop & dairy farm	21	32	18	71/245 (29)
	Shop & sometimes from dairy farm	10	12	1	23/245 (9.3)
	Shop & own cow after calving	0	2	0	2/245 (0.8)
	No milk consumption	0	2	0	1/245 (0.4)
Drink raw milk without boiling	Yes	15	52	23	90 (36)
	No	45	56	56	157 (64)
Report practice of drinking raw milk without boiling	Current	14	53	13	80/245 (32)
	None	44	54	56	154/245 (62)
	Past	1	0	10	11/245 (4.5)
Who drinks raw milk	Adult/s	15	53	23	91/92 (99)
	Children	0	0	0	0
	Adults & children	0	1	0	1/92 (1)
Farm worker only drinks raw milk	Yes	3	45	9	57/90 (63)
	No	12	9	14	33/90 (37)
Method of boiling milk	Boiled, turned off, removed	22	46	1	69/132 (52)
	Boiled for 5–10 min, removed	16	27	11	54/132 (41)
	Boiled for more than 10 min	0	5	4	9/132 (6.9)
Backyard slaughter of cattle	True	24	72	56	152 (62)
	False	36	36	23	95 (38)
Who performs slaughter	Adult male	13	54	38	105/152 (72)
	>1 Adult male	11	15	15	41/152 (28)
	Adult male/s and Children	0	0	0	0
Reason for slaughter	Village festival	12	28	36	76/152 (50)
	Family function	10	33	11	51/152 (41)
	Funeral/Wedding	0	3	1	4/152 (2.7)
	Cattle injury due to road accident	1	3	0	4/152 (2.7)
	Otherwise abnormal or injured cattle	0	2	0	2/152 (1.2)

a Participants selected a single response.

b Indigenous people of the Fiji Islands.

The main water sources reported for households were communal dams (177/247, 72 %). Most households (161/247, 65 %) shared these drinking water sources with cattle. This was particularly so in farm worker (76/108, 70 %) and village (61/79, 77 %) households compared to farm manager households (24/60, 40 %). Multivariable analysis showed that Itaukei households, farm workers and villagers had significantly greater odds of sharing a water source with cattle than did non-Itaukei and farm manager households (Table 3). The interaction between household type and ethnicity was not-significant (*P* = 0.42).

**Table 3:** Multivariable analyses to investigate the association of ethnicity and household type with three known high-risk practices for zoonotic TB among 247 households who owned or worked with cattle in Central Division Fiji in 2018–2019.

Outcome/Variable	Variable categories	P-value	Odds ratio	OR 95 % CI^a^
*Water source shared with cattle*				
Ethnicity	Non-Itaukei		1	
	Itaukei	0.0025	3.03	1.48,6.22
Household type	Farm manager		1	
	Farm worker	0.0068	2.63	1.31,5.31
	Village	0.01	2.90	1.27,6.62
*Drinking raw milk*				
Ethnicity	Non-Itaukei		1	
	Itaukei	0.37	0.71	0.34, 1.49
Household type	Farm manager		1	
	Farm worker	0.0028	3.14	1.48,6.63
	Village	0.37	1.49	0.62,3.55
*Backyard slaughter of cattle*				
Ethnicity	Non-Itaukei		1	
	Itaukei	<0.00001	27.17	10.1, 72.8
Household type	Farm manager		1	
	Farm worker	0.45	1.40	0.59,3.31
	Village	0.69	0.83	0.33, 2.06

a Odds ratio with 95 % confidence interval from logistic regression.

Drinking raw milk without boiling was reported by 36 % of households (90/247). Further exploration suggested that only adults drank raw milk, and that the majority were farm workers (Table 2). Of the participants who boiled milk before drinking it, 44 % boiled and turned off the stove or immediately removed the milk from the stove. In multivariable analysis, drinking raw milk was significantly associated with household type (*P* = 0.002) but not with ethnicity (*P* = 0.37) (Table 3); farm worker households had 3 times greater odds of drinking raw milk compared with farm manager households.

Animal slaughter is a recognised risk factor for *M. bovis* transmission to humans, and 62 % of participants reported backyard slaughter of cattle (Table 2). The practice was primarily performed by adult members of a household and mostly for festivals or for family functions (Table 2). In multivariable models, backyard slaughter of cattle had a significant association with ethnicity (P < 0.00001), with Itaukei households having 27 times greater odds of undertaking this practice compared with non-Itaukei households, but there was no significant association with household type (P = 0.33) (Table 3).

**Fig. 4:**
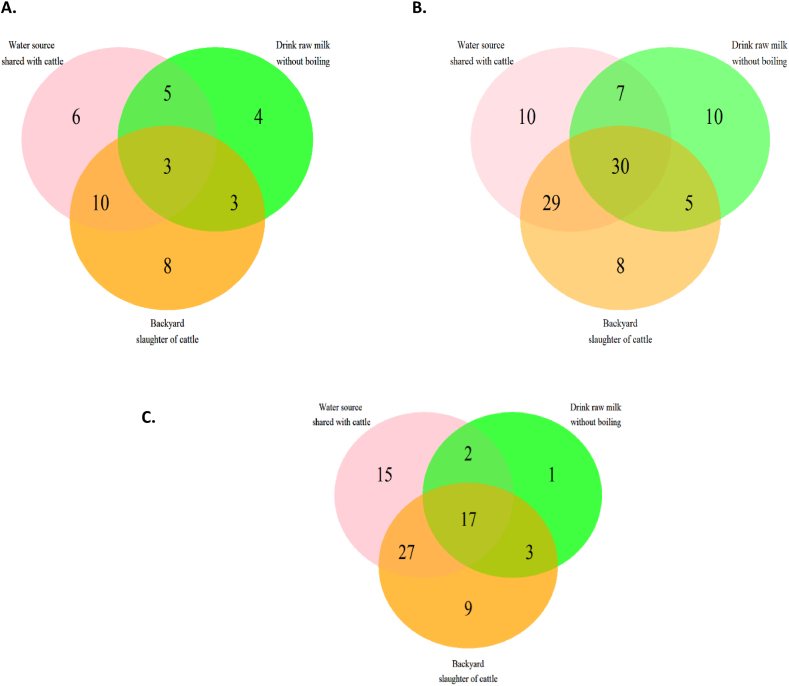
Venn diagrams showing the numbers of households with combinations of risk factors for zoonotic TB among 247 households who owned or worked with cattle in Central Division Fiji in 2018–2019 A. Farm manager households (n = 39). There were another 21 households without these risk factors. B. Farm worker households(n = 99). There were another 9 households without these risk factors. C. Village households (n = 74 in two villages). There were another 5 households without these risk factors.

Farm manager households appeared to be engaged in fewer risky practices. Fig. 4A shows that only three farm manager households were engaging in all three risky practices while 10 identified as having both a water source shared with cattle and performing backyard slaughter of animals. However, farm worker households engaged in multiple practices which posed a risk for zoonotic disease transmission. Thirty farm worker households shared a water source with cattle, drank raw milk and engaged in backyard slaughter of cattle (Fig. 4B). For the villager households, there were 17 undertaking all three risky practices while five had none of these risk factors (Fig. 4C).

#### 3.2.3 Household member level

In total, 1137 household members participated in interviews. Just over a half of the respondents were male. In relation to occupation, 28 % of the participants had work related to dairy farms either as farm managers or farm workers and a similar percentage were students. Few had salaried employment, were retired or unemployed (Table 4). Most respondents had been screened for TB. Almost a quarter (23 %) had regular contact with an infected cattle herd. Around 15 % occasionally drank raw milk and three participants regularly drink raw milk without boiling it. Most participants (94 %) never drank water used by cattle, but most (69 %) swam or played in water used by cattle (Table 4).

**Table 4:** Demographics, zoonotic TB risk practices, and possible TB symptoms among 1137 members of 247 households who owned or worked with cattle in Central Division Fiji in 2018–2019.

Variable	Category	n (%)
Gender	Male	595 (52)
	Female	542 (48)
Occupation	Farmer/Villager - Farm manager farmer/businessman	147 (13)
	Labourer - labourer/airy farm labourer/Dairy farm hand/farm hand	167 (15)
	Domestic duties	266 (23)
	Student - primary/secondary/tertiary	328 (29)
	Child ages 0-5	139 (12)
	Salaried job - policeman, house girl, town job, medical, businessman, cropping	60 (5)
	Retired	17 (1)
	Unemployed	13 (1)
Ever screened for TB	Yes	930 (82)
	No	207 (18)
Known contact with infected cattle herd	Never	431 (38)
	Occasionally	439 (39)
	Regularly	265 (23)
Drink raw milk	Never	965 (85)
	Occasionally	168 (15)
	Regularly	3 (0.3)
Drink water used by cattle	Never	1068 (94)
	Occasionally – less than once a week	61 (5)
	Regularly -once or week or more	6 (1)
Swim/play in water used by cattle	Never	351 (31)
	Occasionally	736 (65)
	Regularly	47 (4)
Current symptoms suggestive of TB	No	1087 (96)
	Yes	50 (4)
Cough >2 weeks	Yes	50/50 (100)
Fever & Night sweats	Yes	14/50 (28)
Unexplained weight loss	Yes	4/50 (8)
Excessive fatigue	Yes	0/50 (0)
Visible neck swelling	Yes	4/50 (8)
Unusual abdominal distension	Yes	0/50 (0)

Only 50 participants (4 %) reported current symptoms potentially suggestive of TB; all reported a chronic cough (>2 weeks duration). In addition to cough, 14 participants reported fever and/or night sweats and four visible neck swelling, suggestive of enlarged cervical lymph nodes (Table 4).

**Fig. 5:**
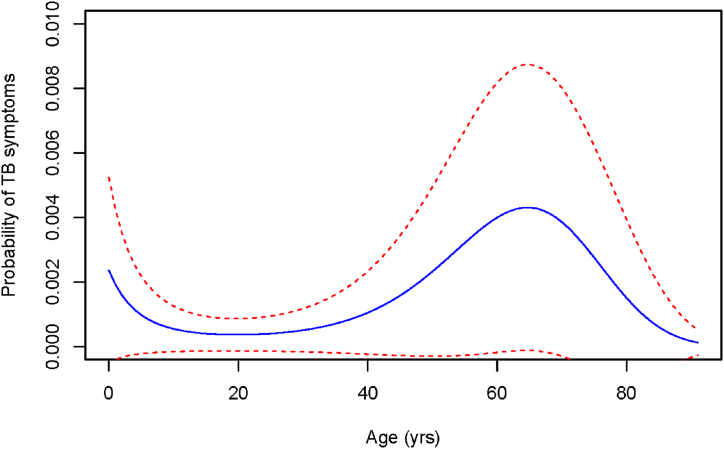
Model-based probability of reporting possible TB symptoms (blue line) with standard error boundaries (red dashed lines) by age for 1137 members of 247 households who owned or worked with cattle in Central Division Fiji in 2018–2019. (For interpretation of the references to colour in this figure legend, the reader is referred to the Web version of this article.)

On univariate analysis age was significantly associated with chronic cough (P < 0.001) with a peak around 60 years of age (Fig. 5). Consistent with this finding people in the retired group had a higher probability of reporting TB symptoms (Table 5). Regularly drinking raw milk without boiling was also associated with higher probability of reporting TB symptoms (Table 5), although there was no association with symptoms specifically suggestive of EPTB (abdominal distension or visible neck swelling). Previous TB treatment was not recorded.

**Table 5:** Univariable analyses of risk factor for reported TB symptoms among 1137 members of 247 households who owned or worked with cattle in Central Division Fiji in 2018–2019.

Variable	P-value^a^	Category	Probability of reporting TB symptoms^b^
Age in years	0.01	See Fig. 5	
Gender	0.26	Female	0.006
		Male	0.009
Occupation	0.04	Farm manager	0.0019
		Farm worker	0.004
		Domestic	0.001
		Student	0.0009
		Child	0.002
		Salary Job	0.0001
		Retired	0.019
		Unemployed	0
Contact with infected herd	0.18	Never	0.004
		Occasionally	0.005
		Regularly	0.012
Drinking raw milk	0.016	Never	0.004
		Occasionally	0.016
		Regularly	0.18
Drinking water used by cattle	0.071	Never	0.006
		Occasionally	0.013
		Regularly	0.26
Swim/play in water used by cattle	0.46	Never	0.001
		Occasionally	0.008
		Regularly	0.031

a P-value in generalised linear mixed model.

b Comparison of probability between categories of variable for reporting of TB symptoms.

## 4 Discussion

This is the first study to report spatial association between human cases of EPTB and cattle with bTB in Fiji. While the occurrence of zoonotic TB among rural communities of Fiji remains to be confirmed microbiologically, it is reasonable to conclude that there is a high risk of transmission of *M. bovis* from cattle to humans in these communities because there is a high prevalence of bovine TB [10,11], there is a large population of people living with or near cattle and because EPTB – a feature of zoonotic TB - has been reported [12,13]. As the first study of its kind in Fiji, it was undertaken to address basic questions about the risk of zoonotic TB in Fiji and was planned and implemented using a One Health approach. There is strong justification for this. In a review of mycobacterial zoonoses, S Thirunavukkarasu, KM Plain, K de Silva, BJ Marais and RJ Whittington [22] identified poor communication between human and animal health experts and entrenched parochialism, stating: “currently, physicians and veterinarians do not engage in productive long-term research collaboration, despite the potential for enhanced insight to be gained”. Operationally, this translates into a lack of zoonotic TB surveillance in the places where it is most needed: almost 90 % of 119 WHO signatory countries had no surveillance for zoonotic TB and an approach in which activities between human and animal health sectors were coordinated was used in only 4 countries (all developed) [23]. Of the 71 countries with bTB, only one in ten looked for zoonotic TB [23].

Acknowledging that the confirmation of zoonotic TB is difficult in the absence of established systems for microbiological and epidemiological investigation and significant resources, this study is an important step to justify future allocation of finite government funds to public health intervention. We utilised existing data on the occurrence and locations of both human EPTB and bovine TB, acknowledging all of the limitations introduced by passive case finding and limited diagnostic capacity and accuracy. The analysis of routine disease data was complemented by new information acquired through face-to-face interviews to assess common risks for exposure of rural populations to cattle harbouring *M. bovis*.

### 4.1 There is a significant spatial association between bovine TB infected farms and human extra-pulmonary TB cases

This study is the first to map the co-location of human EPTB cases and bovine TB infected cattle herds in Fiji. In a recent study, bovine TB was reported to be highly prevalent in cattle, the highest concentration being on dairy farms which are predominantly located in Central Division, which includes Suva, the capital city of Fiji. About 8 % of dairy cattle and 62 % of dairy farms were bTB affected in the period between 2015 and 2020 with a strong concentration of infected dairy farms in Tailevu and Naitasiri provinces of Central Division [11]. Therefore, this risk-based investigation was focused on these two provinces.

The co-location analysis determined that there is a significant spatial association between human EPTB cases and bovine TB infected herds and there was also a visual suggestion of case clustering around farms with high rates/frequency of bovine TB infection. The statistical analysis was limited by the concentration of bovine TB infected herd data points as the population distribution of human EPTB to bovine TB cases was extremely uneven. For the colocation analysis to deem a spatial relationship as significant, the population of human EPTB and bovine TB cases in a spatial relationship must be similar or equal to the actual overall population [17]. With this in mind, if bovine TB cases were less concentrated and the distribution was more widespread throughout the provinces, we would likely see more significant colocations of human and bovine TB cases.

EPTB cases reported by the Fiji TB control programme during the study period, may be inadequate given case detection limitations and the fact that ‘life-long’ EPTB risk should be considered given the long post-infection latency periods observed with zoonotic TB. In addition, there was lack of precision in the designated locations of both bovine TB infected farms (farm gate) and human TB cases (community of residence). The proximity radius approach will be compromised in settings where people who are >5 km away from a dairy farm may still have access to unpasteurised milk or other risk products from the farm – this was not considered in our spatial analysis. Incorporating TB transmission locations would allow for further exploration of TB transmission using a range of spatial analytical techniques that could determine further links between human and bovine cases [24]. For instance, accurate spatial locations of shared water sources between humans and cattle could help determine a spatial transmission point within the Tailevu and Naitasiri provinces [25,26]. Further analysis is warranted to enable a more definitive assessment of spatial association of EPTB in people and bovine TB in cattle in Fiji.

### 4.2 Microbiological evidence of zoonotic TB in Fiji is not available

While *M. bovis* remains the most commonly recognised mycobacterial zoonosis globally, the use of modern tools of molecular epidemiology revealed that zoonotic TB can be due to species other than *M. bovis* [27] and that cattle can become infected with *M. tuberculosis* with risk of spill back to humans [28]. These observations mandate a comprehensive One Health approach but thorough regional surveillance of mycobacterial diseases in all relevant hosts is difficult to achieve. Consistent with the pragmatic approaches adopted for human TB control in many developing countries, human TB diagnosis in Fiji is generally not based on culture or any form of strain typing that differentiates different *M. tuberculosis* complex species or these from other mycobactreria. GeneXpert MTB/RIF is the most widely used test for human case detection and this does not differentiate *M. tuberculosis* sensu stricto from other *M. tuberculosis* complex species such as *M. bovis* or *M. bovis* BCG. Laboratory facilities for species differentiation of *M. tuberculosis complex* do not exist in Fiji, necessitating arrangements with medical laboratories in Australia if cultures are to be performed and analysed. While the Fiji Animal Health Laboratory does undertake routine culture of *M. bovis*, microbiological identification has mostly been presumptive. In 2019, cultures from bovine samples were submitted to laboratories in Australia where *M. bovis* was confirmed using real-time PCR (data not shown). To properly evaluate zoonotic TB in Fiji it will be necessary to undertake more detailed microbiological and molecular epidemiological analyses. This recommendation is consistent with WHO guidelines to improve zoonotic TB diagnosis in people by expanding the availability of diagnostic test capacity [7].

### 4.3 Risk factors for *M. bovis* transmission between cattle

Bovine TB was probably introduced into Fiji with cattle imported by European settlers in the 1830s and has been transmitted ever since. To reduce transmission between cattle and risk to public health risk, an official bovine TB testing programme started in the 1960s, funded by the government of Fiji through the Ministry of Agriculture and was followed in the early 1980's by a combined Brucellosis and Tuberculosis Eradication and Control (BTEC) Program with support from the Australian Government. The components relevant to bovine TB include: 1) dairy farm registration, 2) on-farm testing using the caudal tail fold test for all animals over three months old, 3) slaughter of identified infected animals at abattoirs, 4) abattoir inspection and monitoring of all cattle slaughtered, 5) control of animal movements from infected farms and 6) formal declaration of infected areas [29]. However, a substantial reduction in prevalence of bTB has not been achieved and enhancements have been recommended to achieve adequate control [10,11]. This will be assisted by better understanding of risk factors for bovine TB persistence in Fiji.

Transmission of *M. bovis* and susceptibility to disease in cattle are influenced by biological, behavioural, environmental and genetic factors [30,31]. Herd level risk factors include herd size [32], type of enterprise (dairy > beef) and sharing farm boundaries with infected neighbouring farms [33]. Animal level risk factors include breed, reproductive status, gender, age, body condition score and other health conditions such as helminth infection [33,34]. Cattle movements within a country, as well as animal markets and shows pose major risks for bovine TB spread in a country if infection is not controlled [35], since cattle infect each other through close contact. Cattle may also become infected from contaminated drinking water [36].

The current study investigated farm management practices through a questionnaire, and additional information was available informally from staff of the BTEC program who were in regular contact with registered farms, received updates about the farms’ bovine TB status and had opportunities to observe ongoing farm management practices; they were well placed to provide clarification on current practices. Factors that should be considered in future studies include the failure of bTB diagnostic test protocols to identify all infected individuals, movements of infected cattle between farms, contact between different herds on shared community pastures, inadequate fencing between farms, transmission of *M. bovis* between other susceptible domestic species on farms and possible wildlife reservoirs for *M. bovis*.

While most of the participants grazed their cattle within a fenced paddock, they also allowed them to roam freely, which agrees with the findings reported by W Ayele, S Neill, J Zinsstag, M Weiss and I Pavlik [37] and M − F Humblet, ML Boschiroli and C Saegerman [38]. According to BTEC staff, on most farms cattle roamed freely, mainly within the boundaries of their farms, but in some areas the cattle grazed in the hills during the morning and were brought back to the farm during the evening for milking. Dry cows were allowed to stay in hills until they were ready to mate. According to BTEC staff, the farms with infected status had farm boundary markers, but they did not have proper fencing. Farms with a Bovine TB clear status appeared to be more cautious in securing their boundary fences. At the time of writing (2022), only two farms were able to maintain a bovine TB clear status, while the rest had either been reinfected or latent cases had emerged during ongoing annual testing [11]. Free roaming for either a part of a day or a full day significantly increases the interaction of cattle from different herds, which increases the risk of contact with *M. bovis*, and this may explain the breakdown of clear status over time.

Free roaming also increases the likelihood of cattle coming into contact with wildlife. Cattle are the maintenance host for *M. bovis* but the infection is extremely difficult to eradicate in domestic species once it has become established in a wildlife reservoir [33,39]. In many countries wildlife are reported to be a reservoir for *M. bovis* and are described as a major risk factor [40]; the species of wildlife reservoir host varies between countries. There has been one study of wild Mongoose (*Herpestes auropunctatus*) in Fiji. They have both a high population density and frequent contact with cattle on farms in Fiji, but seem unlikely to be a reservoir host for *M*. *bovis* [41]. This is despite the anecdotal evidence from Fiji that mongooses scavenge dead cattle and published reports of *M. bovis* in South Africa in the banded mongoose (*Mungos mungo*) [42] and in Portugal in the Egyptian mongoose (*Herpestes ichneumon*) [43]. Better understanding of possible wildlife reservoirs in Fiji and cattle grazing practices will provide information for the development of programs that integrate both local and scientific knowledge for bovine TB control in cattle and zoonotic TB prevention in human populations living at the wildlife-livestock interface.

Beyond grazing of common pastures, using a shared water source is considered a possible risk factor for *M. bovis* transmission between cattle [33,36]. In this study there was a significant difference in the proportions of farms with a clear or infected status associated with grazing over unfenced dams or springs, but the effect was the opposite of that expected, i.e. there was a higher proportion of clear farms than infected farms with this feature. Furthermore, herd size was not significant, whereas it is known to be based on a larger study in Fiji [11], and as reported by others [33]. The difference between these studies probably relates to the frequencies of farms in each bovine TB status category being inaccurate. Most of the clear farms were classified as infected in subsequent rounds of bovine TB testing, as reported elsewhere [11]. Clearly there is a need for further investigation in Fiji to provide reliable evidence to inform recommendations about grazing management. Interventions such as fencing farm boundaries, preventing access of cattle to common dams and creeks and provision of alternative water sources, which are regarded as important principles of bovine TB control in other countries, is likely to meet farmer resistance and would be very expensive to implement in Fiji.

### 4.4 Risk factors for *M. bovis* transmission between cattle and humans

Risk factors for M. bovis transmission to people include food sources and eating habits, living in close contact with animals such as sharing the same shelter for sleeping, occupation, socio-economic status, customs and traditions, and acquired immunodeficiency due to HIV/AIDS [37,44,45].

Human infection with *M. bovis* is a risk wherever bovine TB exists. *M. bovis* infection is primarily acquired through the consumption of unpasteurised milk and dairy products and less frequently from eating raw or improperly cooked infected meat or rarely via aerosols inhaled from infected animals during direct human–livestock contact [46,47]. The consumption of raw (unpasteurised) milk and animal slaughtering have been identified as major risks for *M. bovis* transmission from animals to humans [[47], [48], [49]]. In the current study, 35 % of households reported that they consumed raw milk. The investigation revealed that farm worker households were exposed to the greatest risk, more so than the households of farm owners or managers. The same was true for another potential risk factor for zoonotic TB, households sharing water sources with cattle, given that *M. bovis* can transmit between cattle in this way [36].

Demographic characteristics at least partially influenced these risks in Fiji, with the Itaukei ethnic group being more likely to conduct backyard slaughter and to have a water source common with cattle; they are primarily farm workers and are based in villages. These findings are linked to the socio-economic status of the ethnic groups as reports show that Indo-Fijians are more likely to be seen in business and generally have higher economic status than indigenous Fijians [50]. Note that non-Itaukei farm manager and/or worker households likely would not have conducted backyard slaughter of potentially *M. bovis* infected cattle because of religious beliefs that consider cattle as sacred.

At the household member level, participant age showed the strongest correlation with reported TB symptoms. Older male participants who were retired reported the highest frequency of chronic cough, which is associated with a TB diagnosis (less commonly with EPTB) but also highly correlated with years of tobacco smoking. Smoking status was not recorded in this study and older people have many other conditions that should be considered in the differential diagnosis of symptoms potentially associated with TB. Apart from current ‘TB symptoms’, documentation of past TB treatment, especially for EPTB, might provide a more accurate measure of lifetime zoonotic TB risk. Children in Fiji receive routine BCG vaccination, which provides a level of protection against zoonotic TB, although the strength and duration of the protective effect has not been measured [51].

There is a potential role for the environment to maintain *M. bovis* and lead to infection of domestic cattle, wild animals or humans. The environment, particularly water and soil, can act as a reservoir for *M. bovis,* although the dynamics of transmission is complex [30,52]. The environment is considered to be an important reservoir for maintaining infection in multi-host systems and can facilitate indirect transmission via different routes [52,53]. In natural conditions *M. bovis* can survive up to 88 days in soil, 58 days in water and for 43–58 days in feeds such as hay and corn [54]. Neglecting the potential environmental risk may undermine efforts to control *M. bovis* infection in both humans and animals [55]. The current study revealed dairy farm practices, for example sharing natural water resources like rivers and creeks and grazing cattle on communal land, that could result in environmental contamination. There has been no direct investigation of the environment as a potential contributor to *M. bovis* transmission in Fiji but this is needed to inform prevention strategies for bovine TB and to prevent zoonotic TB in Fiji.

### 4.5 Farmer knowledge to prevent zoonotic TB and control bovine TB is inadequate

Although not formally investigated in this study, the findings demonstrate that the farmers had limited understanding of the practices required to prevent bovine TB at the farm level. Even after the effort of obtaining a bTB clear disease status through an expensive test and cull program they continued to engage in practices that posed a risk for disease transmission to animals, as well as to humans. Farm workers (including village farmers) generally placed themselves at greater risk of contracting *M. bovis* infection than did farm owners and managers. These findings are not dissimilar from those in a study in Bangladesh, where almost 60 % had insufficient knowledge about *M. bovis* transmission to humans and risky practices such as sharing the same premises with animals were common [56]. A targeted education/awareness approach is required in Fiji to reduce the future risk of zoonotic TB and to ensure uptake of recommendations and practices aimed at controlling and preventing bovine TB. Change is needed at both farm and household levels.

## 5 Conclusions and recommendations

Despite the low quantity of statistically significant associations, human EPTB cases in Fiji appear to be over-represented in places where there are bovine TB infected cattle farms. Further analysis is warranted to enable a more definitive conclusion on the spatial association between human EPTB disease and *M.bovis* infected farms in Fiji. Data should be evaluated over a longer time period (>15 years) with appropriate consideration of risk and disease measures and include lifetime EPTB diagnoses as well as farm and household location verification by ‘ground-truthing’. The potential for long latency of TB is a problem for all such studies especially considering the potential for migration of farm workers to other geographical regions in Fiji. Therefore, an assessment of the mobility of farm workers (between farms, districts and industries) over their working lives and analyses that can adjust for this would be important in future studies.

This information would be useful for objective assessment of the risk for zoonotic TB occurrence in the country, and to measure progress in tackling TB in both cattle and humans. A One Health approach is necessary for comprehensive investigations of zoonotic TB [57,58]. A commitment is required to test all EPTB cases based on risk factor stratification, to confirm all "true" *M. bovis* human TB cases. The potential role of environmental reservoirs in water and soil to transmission of *M. bovis* also requires thorough investigation.

There are no microbiological data to confirm or rule out zoonotic TB in Fiji and to properly evaluate this in Fiji it will be necessary to establish procedures for appropriate specimen collection and mycobacterial speciation, from both human and animal specimens. The existing laboratory protocols for mycobacterial culture from clinical samples from cattle at the Fiji Veterinary Laboratory of the Ministry of Agriculture should be enhanced to enable definitive identification of disease-causing mycobacteria with archiving. This essential work could be conducted in Fiji after building the necessary human capacity and laboratory infrastructure, or it could be outsourced. This investigation will require funding, coordination, detailed planning, training of personnel, increased laboratory capacity and agreements with international partners.

Further investigation should be undertaken in Fiji to inform recommendations about grazing management, specifically the need for fencing of farms and keeping cattle out of village water sources used by people. As the BTEC program has continued to the present time in Fiji [11], there is a good opportunity to evaluate bovine TB risk factors by farm TB status. Classification of farm status is likely to be more accurate today than it was in 2017 due to ongoing testing and the use of a central database [11]. Interventions such as fencing farm boundaries, preventing access of cattle to common dams and creeks and provision of alternative water sources, which are known to be important principles to control bovine TB in other countries, would be costly for Fijian farmers to implement and so must be based on evidence. This will be essential for willing adoption of recommendations by farmers and for government support. These interventions would also reduce the risk of zoonotic TB.

Farmers had limited understanding of the practices required to prevent bovine TB at farm level and some continued to engage in practices that posed a risk for disease transmission after their cattle tested negative for bovine TB. A targeted education/awareness approach is required to reduce the future risk of zoonotic TB and to help ensure uptake of recommendations and practices aimed at controlling and preventing bovine TB. Change is needed at both farm and household levels, and needs to be based on ethnically-relevant programs informed by social scientists due to the risk differences observed between ethic groups in this study.

## Ethics

Ethics approval for this activity was obtained from the 10.13039/501100001774University of Sydney Human Research Ethics Committee (Protocol no. 2019/648) and the Fiji National Human Research and Ethics Review Committee (Protocol no. 2019.86.NW).

## Data availability statement

Data will be made available on request.

## CRediT authorship contribution statement

**Jenny-Ann L.M.L. Toribio:** Conceptualization, Formal analysis, Funding acquisition, Investigation, Methodology, Project administration, Resources, Supervision, Validation, Visualization, Writing - original draft, Writing - review & editing. **Keresi Lomata:** Conceptualization, Investigation, Methodology. **Sam Fullman:** Investigation, Methodology. **Aaron Jenkins:** Conceptualization, Data curation, Formal analysis, Methodology, Writing - original draft, Writing - review & editing. **Elva Borja:** Conceptualization, Methodology, Writing - original draft, Writing - review & editing. **Shumaila Arif:** Data curation, Formal analysis, Visualization, Writing - original draft, Writing - review & editing. **Jarrad McKercher:** Data curation, Formal analysis, Methodology, Writing - review & editing. **David Blake:** Formal analysis, Methodology, Writing - original draft, Writing - review & editing. **Anabel Garcia:** Data curation, Formal analysis. **Richard J. Whittington:** Formal analysis, Validation, Visualization, Writing - original draft, Writing - review & editing. **Frank Underwood:** Conceptualization, Investigation, Methodology. **Ben J. Marais:** Conceptualization, Methodology, Writing - original draft, Writing - review & editing.

## Declaration of competing interest

The authors declare that they have no known competing financial interests or personal relationships that could have appeared to influence the work reported in this paper.
