# Supplementary material for: Assessing risks for bovine and zoonotic tuberculosis through spatial analysis and a questionnaire survey in Fiji – A pilot study
Source: Heliyon. 2023 Nov 23;9(12):e22776. doi: 10.1016/j.heliyon.2023.e22776 (PMC10730600; doi:10.1016/j.heliyon.2023.e22776)
Supplement: Multimedia component 1 [file mmc1.docx]

| Sub-division / Province |  | Nearest Health Facility: |  |
| --- | --- | --- | --- |
| Farm ID or Community ID: |  | **PUT enough detail to be able to FIND RESIDENCE** | |
| Household ID: |  | Household Address/Phone: |  |
| Household Surname: |  | Residence GPS coordinants |  |
| Date of interview |  | Name of interviewer: |  |

**Household Head Interview** Name:

**Current Household^[[1]](#footnote-1)^ and TB Screening in this program ^[[2]](#footnote-2)^RETURN TO ASK LATER – Pg 4**

| **Household** | **Age (yr)** | **Gender** | **Occupation** | **Screened for TB** | **Contact with infected cattle herd** | **Drink raw milk** | **Drink water used by cattle** | **Swim/play/contact water used by cattle** |
| --- | --- | --- | --- | --- | --- | --- | --- | --- |
| Member 1  Name |  |  |  | Yes / No | R / O / N | R / O / N | R / O / N | R / O / N |
| Member 2  Name |  |  |  | Yes / No | R / O / N | R / O / N | R / O / N | R / O / N |
| Member 3  Name |  |  |  | Yes / No | R / O / N | R / O / N | R / O / N | R / O / N |
| Member 4  Name |  |  |  | Yes / No | R / O / N | R / O / N | R / O / N | R / O / N |
| Member 5  Name |  |  |  | Yes / No | R / O / N | R / O / N | R / O / N | R / O / N |
| Member 6 Name |  |  |  | Yes / No | R / O / N | R / O / N | R / O / N | R / O / N |
| Member 7  Name |  |  |  | Yes / No | R / O / N | R / O / N | R / O / N | R / O / N |
| Member 8  Name |  |  |  | Yes / No | R / O / N | R / O / N | R / O / N | R / O / N |

**Any household member with current symptoms suggestive of TB?**

| **HH member**  **(List No from above)** | **Cough >2 weeks** | **Fever & Night Sweats** | **Unexplained weight loss** | **Excessive fatigue** | **Visible neck swelling** | **Unusual abdominal distension** |
| --- | --- | --- | --- | --- | --- | --- |
|  |  |  |  |  |  |  |
|  |  |  |  |  |  |  |
|  |  |  |  |  |  |  |
|  |  |  |  |  |  |  |
|  |  |  |  |  |  |  |

**Household TB Disease History**

In the last 5 years - Have any current or past members of the household had TB? **Yes / No**

If YES – please provide history on

| **HH member**  **(List No from above)** | **Year at diagnosis** | **Age at diagnosis** | **Gender** | **Occupation** | **Type of TB (PTB, EPTB)** | **Year treated** | **Outcome** |
| --- | --- | --- | --- | --- | --- | --- | --- |
|  |  |  |  |  |  |  |  |
|  |  |  |  |  |  |  |  |
|  |  |  |  |  |  |  |  |

In the last 5 years – Have any family contacts or close/house friends been treated for TB (including people who may now be deceased)? **Yes / No**

If YES – please provide history on

| **Family/friend** | **Year at diagnosis** | **Age at diagnosis** | **Gender** | **Relationship to HH** | **Occupation at diagnosis** | **Location of residence** | **Type of TB** | **Year treated** | **Outcome** |
| --- | --- | --- | --- | --- | --- | --- | --- | --- | --- |
| Person 1 |  |  |  |  |  |  |  |  |  |
| Person 2 |  |  |  |  |  |  |  |  |  |
| Person 3 |  |  |  |  |  |  |  |  |  |
| Person 4 |  |  |  |  |  |  |  |  |  |

**Type of TB: PTB Pulmonary tuberculosis, EPTB Extra-pulmonary tuberculosis**

In the last 5 years – Have any family contacts/ close house friends died of an unexplained disease associated with weight loss, neck swelling or abdominal distention? **Yes / No**

If YES – please provide history on

| **Family/friend** | **Year at death** | **Age at death** | **Gender** | **Relationship to HH** | **Occupation** | **Location of residence** |
| --- | --- | --- | --- | --- | --- | --- |
| Person 1 |  |  |  |  |  |  |
| Person 2 |  |  |  |  |  |  |
| Person 3 |  |  |  |  |  |  |

**Potential contacts for household members with infected cattle, raw milk or contaminated water**

| What is the main source of water used by the household? |  |
| --- | --- |
| Are any other water sources used by the household? | Yes / No  **If Yes - specify** |
| Are any of these water sources shared with cattle? | Yes / No  **If Yes - specify** |
| What is the usual sources of milk used by the household? |  |
| Does anyone in the household drink milk fresh from the cow without boiling it? | Yes / No  **If Yes – List WHO in household drinks raw milk** |
| For household that boils milk –  **WHAT is the process used for boiling?** **Describe** |  |
| Does the household participate in any backyard slaughter of cattle? | Yes / No  **If Yes-List WHO in household helps with slaughter** |
|  | **WHY** is backyard slaughter done? |
|  | **WHEN** was the last backyard slaughter?  **Month / Year** |

**Additional Questions for Household that raises / owns cattle**

1.What type of cattle do you raise? Dairy; Beef; Both

2. What is the purpose of cattle raising? Tick all that apply

□ Main income source

□ Secondary income source

□ Household consumption of milk

□ Household consumption of meat

□ Other - Please specify:_____________________________

3.Current number of cattle

| Calves  (<6 months old) | Cattle  (not for breeding) | Cows for breeding | Bulls for breeding |
| --- | --- | --- | --- |
|  |  |  |  |

4.How are your cattle managed? Tick all that apply

□ Grazing pasture in fenced paddocks

□ Tethered at all times

□ Held in stall or yard at all times with cut-and-carry feeding

□ Roam freely for grazing

5.What water sources do your cattle access? Tick all that apply

□ River/creek

□ Well/spring

□ Dam

□ Water trough or other containers

□ Other - Please specify:_____________________________

Which of these is the main water source for cattle? ___________________________________

6.In the area/s that your cattle graze, are any of the following present? Tick all that apply

□ River/creek

□ Unfenced Dam

□ Unfenced Well or Spring

□ Cattle owned by other people

□ Wild or unowned cattle

□ Other - Please specify:_____________________________

7.Where are your cattle slaughtered?

□ Abattoir

□ On the farm

□ At the residence

□ Other - Please specify:_____________________________

8.Have your cattle been tested for cow TB infection before? Yes / No

If Yes – What was the date of the last test? _____________ Result? ________________

1. Household refers to all people living at the residence for the last 6 months or longer. [↑](#footnote-ref-1)
2. Regularly – once a week or more; Occasionally – less than once a week; Never – Not at all [↑](#footnote-ref-2)
